# Detection of mutations: from Ames test to duplex sequencing

**DOI:** 10.3389/fmolb.2026.1774439

**Published:** 2026-04-02

**Authors:** Niketa Bhawsinghka, Roel M. Schaaper

**Affiliations:** Genome Integrity and Structural Biology Laboratory, National Institute of Environmental Health Sciences, Durham, NC, United States

**Keywords:** Ames assay, duplex sequencing, error-corrected next-generation sequencing, mutagen, mutation, mutation spectra

## Abstract

Mutation is a biological phenomenon observed in all life forms from viruses to humans. This inescapable process has fascinated scientists for nearly a century. Mutagenicity has become a concern since the 1940s following the discovery that chemicals can cause mutations, because of which the scientific community has ventured into finding effective methods of detecting harmful mutagens. The earlier studies in this field were carried out using organisms like *Escherichia coli*, *Drosophila*, and *Neurospora*. Later, the breakthrough development of an assay using bacteria allowed researchers to detect the abilities of chemical compounds or mixtures to induce DNA mutations. This assay came to be named as the Ames test after its developer Bruce Ames; since then, it has been widely adopted for mutagenicity testing. The introduction of Sanger sequencing technology enabled researchers to explore beyond phenotypic changes and uncover detailed information on DNA sequence changes and mutational spectra. With the advent of next-generation sequencing (NGS), it has become possible to expand mutation analysis to the larger genome without the need for phenotypic selection, particularly given the development of various error-corrected NGS (ecNGS) techniques. Duplex sequencing (DS) is a relatively new ecNGS technique that can detect mutations at low frequencies in isolated DNA. In this mini review, we briefly explore the genetics of the Ames test and shed light on DS as an emerging tool for detecting mutations.

## Introduction

Mutation is a multi-affair process that can result from errors in DNA metabolism as well as environmental stresses. The frequency of mutations generally remains low (Drake et al., 1998; Cervantes et al., 2002) owing to the operations of multiple pathways responsible for maintaining genome integrity (Schaaper, 1993). Mutations have intrigued mankind starting from the early 1900s. Auerbach and Robson (1946) reported the first chemical mutagen, namely, mustard gas; in the years following this discovery, scientists became interested in understanding the detailed properties of environmental mutagens and their reactions with DNA, which led to numerous developmental efforts toward mutation detection and mutagen testing.

As a pioneering scientist in this field, Bruce Ames isolated several *Salmonella typhimurium* strains to characterize the histidine operon and used them to identify mutagens (Ames, 1979; Ames et al., 1973a; Ames et al., 1973b; McCann and Ames, 1975). However, the use of bacteria for identifying mutagens precedes Ames (Zeiger, 2019). Early studies using *Escherichia coli* monitored the appearance of streptomycin-resistant mutants (Demerec, 1951) or T-phage-resistant mutants (Scherr et al., 1954) as markers to detect mutations. Ames believed that microorganisms were a useful system for detecting mutagens because they react with DNA, which has the same four bases as building blocks arranged in the Watson–Crick structure in all living things (Ames, 1972). This assay developed by Ames later came to be known as the Ames test.

Around the same time, the advent of DNA sequencing and particularly Sanger sequencing (Sanger et al., 1977) offered greater insights into the multitudinous nature of mutations, revealing the contributions of multiple classes, such as base substitutions, frameshifts, indels, duplications, transposable elements, and complex events. Mutational spectra or “fingerprints” obtained by DNA sequencing provide insights into the precise nature of DNA-damage-induced mutations, including the site, sequence, and strand preferences (Coulondre and Miller, 1977; Douglas et al., 2025; Garibyan et al., 2003; Kozmin et al., 2019; Oller et al., 1992; Schaaper et al., 1990). In parallel, important information was obtained on mutations in normally growing cells, or “spontaneous” mutations (Schaaper, 1993; Fijalkowska et al., 1997; Fijalkowska et al., 2012; Foster et al., 2015; Fowler and Schaaper, 1997; Maki and Sekiguchi, 1992; Maslowska et al., 2018; Miller et al., 2002; Niccum et al., 2020; Tse et al., 2016); these not only provide a background for any chemically induced mutations but are also significantly relevant to human health.

Several reporter-based mammalian mutagenicity assays have also been developed to detect mutations *in vitro* (e.g., HPRT, XPRT, and TK assays), which are based on the functional inactivation of genes and detection by selective agents (DeMarini et al., 1989). Subsequently, the introduction of genome-manipulation methods have enabled incorporation of artificial reporter genes into mammalian genomes, giving rise to transgenic rodents (TGRs) that enable detection of mutations *in vivo* (Dycaico et al., 1994). The commonly used TGR systems (BigBlue and MutaMouse) have bacteriophage vectors integrated in tandem arrays within the rodent genome, which are introduced into bacterial hosts after recovery from exposed rodents. Under selective agents, the mutant vectors produce specific plaques or colonies. *In vitro* systems are useful for detecting mutations in mammalian cells but are limited by tissue type, expertise, and time-intensive cell culture methods. However, TGR assays provide flexibility with regard to diverse tissue types but the entire procedure is time consuming, expensive, laborious and requires sacrifice of animals.

The invention of next-generation sequencing (NGS) has expanded the avenues of mutation research. This deep sequencing technology enables analysis of individual DNA fragments rather than accumulated sequences. However, conventional NGS is not particularly useful for identifying mutations given the high background (DNA polymerase) errors (10^−2^ to 10^−3^) (Glenn, 2011). More recently, numerous error-correction strategies have been developed to reduce or even eliminate NGS errors and are referred to as error-corrected NGS (ecNGS) (Flaherty et al., 2012; Kozarewa et al., 2009; Marchetti et al., 2023; Salk et al., 2019). Among these, duplex sequencing (DS) is a well-developed technique that has important applications for both basic research and mutagen testing. In the present review, we highlight two different methods for mutation detection, namely, the Ames test and DS, which are both transformative advancements in the field.

## Evolution of the Ames test

The Ames test is a bacteria-based reverse mutation test, which assumes that the mutagenic activity in bacteria is predictive of the mutagenic activity in humans (Ames, 1971). This simple and convenient assay has been in use for almost 40 years. Many different classes of compounds have been identified as mutagenic using this assay (Furuhama et al., 2025; McCann and Ames, 1975). There is a large body of literature on the Ames test, including several excellent reviews that detail its methodology and applications as well as its use in regulatory testing (Mortelmans and Zeiger, 2000; Zeiger, 2019). Here, we discuss the genetic modifications of the *Salmonella* strains used in the Ames test, which led to the evolution and enhanced the sensitivity of the test.

The assay uses various mutant strains of *S. typhimurium* that are unable to synthesize the amino acid histidine (*his* ^−^). The assay initially used four strains: TA1530 (*his*G46Δ*uvrB*Δ*gal*), TA1531 (*his*C207Δ*uvrB*Δ*gal*), TA1532 (*his*C3076Δ*uvrB*Δ*gal*), and TA1534 (*hisD*3052Δ*uvrB*) (Ames, 1972). The TA1530 strain detects base pair (bp) substitutions by direct reversal of the *his*G46 mutation, while the other three strains detect short (1–2 bp) indels in *hisC* or *hisD* genes. A positive test is indicated by reversion (*his* ^*-*^ *→ his* ^*+*^) to histidine independence. A defective DNA nucleotide-excision repair system (Δ*uvrB*) was introduced to block nucleotide excision and increase the detection sensitivity (Ames, 1971). The Δ*uvrB* alleles were isolated independently as mutations conferring anaerobic resistance to chlorate (Ames et al., 1973b); they each carried a deletion of a different length through the *gal*, *bio*, *uvrB*, and *moa* operons (Porwollik et al., 2001). Among these, the latter is essential for synthesis of the molybdenum cofactor (Moco) and is the basis for chlorate resistance. Moco deficiency additionally increases the possibility of enhanced mutagen susceptibility (Porwollik et al., 2001). Further sensitization was conferred through impairment of the lipopolysaccharide (LPS) layer that acts as an entry barrier for many chemicals. A partial deletion of the *gal* operon (Δ*gal*) prevented incorporation of galactose into the LPS layer, compromising its integrity. Further sensitivity increases were achieved by adding deep-rough mutations (*rfa*) through selection for resistance to phage C21, which shrank the LPS layer to its ketodeoxyoctanoate lipid core (Ames et al., 1973b). The new strains were designated as TA1535 (*his*G46Δ*uvrBrfa*), TA1536 (*his*C207Δ*uvrBrfa*), TA1537 (*his*C3076Δ*uvrBrfa*), and TA1538 (*hisD*3052Δ*uvrBrfa*). Subsequently, TA1535 and TA1538 were transformed with plasmid pKM101 (McCann et al., 1975) to improve their sensitivity to mutations via the error-prone SOS pathway, which resulted in TA100 [*his*G46Δ*uvrBrfa* (pKM101)] and TA98 [*hisD*3052Δ*uvrBrfa* (pKM101)]. These genetic modifications and the incorporation of liver homogenates for metabolic activation of compounds (Ames et al., 1973a) have played important roles in the development of the Ames assay, resulting in its widespread use and inclusion in mandatory regulatory testing (OECD471, 2020). The TA1535, TA1537, TA98, and TA100 strains are four out of five strains recommended by the OECD471 guidelines because of their reliability and reproducibility in detecting mutations at GC sites, while the TA102 [*his*ΔG8476*rfa(pAQ1/pKM101*)] strain (Levin et al., 1982) is specifically designed to detect mutations at AT sites.

Bacterial systems like the Ames assay are relatively easy, time-saving, and cost-effective procedures; however, they are far simpler than human systems, which could raise issues regarding validity. Substances yielding bactericidal outcomes are not suitable for assessment using this test, while phenotypic endpoints may not always be achieved by mutations at the expected target sites. Importantly, the limited target sites provided by the assay are likely not indicative of many events occurring throughout the genome. In contrast, NGS technologies, particularly ecNGS, have the potential to overcome these limitations because the mutations are detected at the sequence level and do not rely on phenotypic outcomes. The newer technologies allow global mutation analysis and can be applied to DNA isolated from any organism, thus eliminating the need for extrapolation.

## DS: an ecNGS technology

NGS has introduced significant changes in genetics and medicine. The massive and parallel sequencing of DNA fragments enable rapid access to the entire genome of an organism and present unique opportunities to identify minor or rare mutants. However, the high error rates of DNA polymerases used in NGS (10^−2^ to 10^−3^) preclude the detection of many or most mutations. This is further constrained if one wishes to detect mutations in a mixture of genomes. Detection above the background requires clonal selection of mutants or mutation accumulation experiments, which are time-consuming and may introduce artifacts (Kanagawa, 2003; Meyerhans et al., 1990). To improve the accuracy of NGS, several approaches have been employed, such as modifications to the library preparation (Chen et al., 2017), statistical modeling of error profiles (Wei et al., 2011; Wilm et al., 2012), bioinformatic filtering (Weissbach et al., 2021), and consensus-based error correction (Hiatt et al., 2010) (ecNGS). Among these, the consensus-based error-correction strategies have proven to be the most significant approaches (Salk et al., 2019). A comparative review of various ecNGS methods is beyond the scope of the current review but is available in a number of other excellent reviews (Salk and Kennedy, 2020; Salk et al., 2018; Sloan et al., 2018; Yauk et al., 2026). Here, we focus on the ecNGS technique known as DS. This method was invented approximately 13 years ago (Schmitt et al., 2012) and has shown significant potential in advancing mutagenesis research, including efforts toward supporting inclusion of DS in regulatory testing.

**FIGURE 1:**
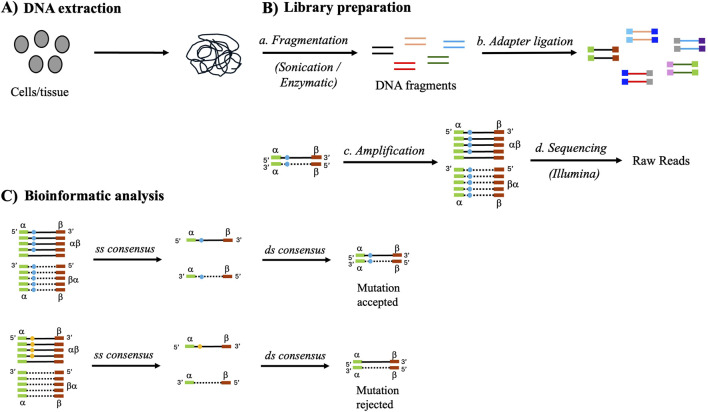
Schematic overview of the duplex sequencing technique involving three major steps. **(A)** DNA extraction from the desired cells or tissues. **(B)** Library preparation: the extracted DNA is fragmented into 200–300-bp fragments by either mechanical or enzymatic methods. After end repair and A-tailing, the fragments are ligated to adapters at both ends, with each fragment having a unique pair of ligated adapters. The adapter-ligated fragments are subjected to polymerase chain reaction amplification to generate many copies, followed by Illumina sequencing. **(C)** Raw reads are bioinformatically processed and two single-strand (ss) consensus sequences are derived, which are subsequently used to form a double-strand (ds) consensus sequence. If a mutation is legitimate and present in both strands (depicted by blue closed circle), it will be incorporated in all or at least a majority of the family members of both strands, appearing in the two ss consensus sequences and ultimately in the ds consensus sequence to result in an accepted true mutation. However, if a mutation arises because of a ss DNA damage (depicted by yellow closed circle), it will be present in only the family derived from the damaged strand and associated ss consensus sequence; owing to its absence in the other ss consensus sequence, the mutation will be rejected.

DS is a consensus-based error-correction technique capable of detecting low-frequency mutations with high accuracies (Schmitt et al., 2012); mutation frequency is defined as the ratio of the number of mutant base pairs to the total number of base pairs sequenced. DS relies on tagging and sequencing each of the two strands of unique DNA duplex fragments (Figure 1). These fragments are ligated with duplex DNA adapters containing degenerate tags or unique molecular indices (UMIs) that serve as short barcodes, which allow reconstruction of the original double-strand DNA after the two strands have been separated during amplification. Amplification is performed to generate many copies of each DNA fragment, and the products are subjected to short-read Illumina sequencing. To examine specific target regions, the amplified fragments can be enriched by prior hybridization with biotinylated oligonucleotides. The sequencing reads are then processed bioinformatically and aligned to a reference (Bhawsinghka et al., 2023; Valentine et al., 2020). The aligned reads are grouped into families based on identical tags (or a combination of tags and unique shear points), and sequences within the families are combined to form single-strand consensus (SSC) sequences. Two complementary SSC sequences (identified by complementary UMIs) are then used to generate a double-strand consensus (DSC) sequence, which is inspected for mutations. A *bona fide* mutation is accepted in the DSC only if the identical mutation is present in both SSCs. In this manner, spurious DNA polymerase errors and other random sources of mutations can be filtered out as they are generally present in only one of the strands (Figure 1) (Kennedy et al., 2014).

A key aspect for identifying mutations by DS is obtaining an appropriately high number of sequencing reads. For example, to identify a mutation at the low level of 10^−8^ per bp, the total number of sequenced bases would have to be at least 10^8^. Using a typical genomic mutation detection target of 10,000 bp would mean that approximately 10^4^ such individual targets would need to be sequenced. This corresponds to each bp being read 10,000 times (duplex depth). Since the DNA is ultimately sequenced in small fragments (∼200-bp size), reading 10^8^ bp would require approximately 5 × 10^5^ small fragments, corresponding to 5 × 10^5^ sequencing “reads.” In reality, the actual number needs to be significantly higher because DS allows acceptance of mutations only when recorded in families of sufficient size. The recommended number for this family size is a minimum of six (with three read pairs from each strand), but larger numbers would offer better results. Optimal family sizes can be obtained by adjusting the DNA input prior to amplification, which is mostly done empirically. Very low DNA inputs would result in large family sizes but fewer families (reducing the total number of scored mutations), whereas very high DNA inputs will generate many more families but of smaller size, many of which may no longer meet the acceptance criteria. For experiments with bacterial DNA using a sequencing target of 10,000 bp, we typically apply 0.8 ng of input DNA that yields approximately 50 M reads (Bhawsinghka et al., 2023); however, for mammalian applications with a 48,000 bp target, approximately 500 ng of input DNA may be used to yield approximately 250 M reads (LeBlanc et al., 2022; Dodge et al., 2023). In both cases, a sequencing depth of 10,000–15,000 is achieved, allowing detection of mutations approaching 10^−8^ in principle; however, this greatly depends on the specific type of bp substitution. The quality of the DNA preparation is a major impediment to achieving low mutant frequencies. In particular, G·C→A·T transitions as well as G·C→C·G and G·C→T·A transversions are observed at high frequencies, often in the 10^−7^ range because of DNA damage during library preparation. The amount of input DNA to be used is otherwise determined by the efficiencies of factors like adapter ligation and target enrichment, further limiting the final duplex recovery fraction to the range of 1%–10% (Kennedy et al., 2014). Hence, detecting just one mutation at the 10^−8^ level might require approximately 50 million reads per sample in the bacterial example and approximately 300–500M reads in the mammalian example. Thus, although DS is accurate and sensitive, it might place a strain on the resources depending on the goal. Nevertheless, since its inception, DS has been used for various applications as described below.

## Applications of DS: from fundamental research to genotoxicity testing

The DS technique was developed in Dr. Lawrence Loeb’s laboratory and was first reported in 2012 (Schmitt et al., 2012). It was first applied to detect mutations in bacteriophage M13 DNA and produced a frequency of 2.5 × 10^−6^ per nucleotide, which was similar to the 3 × 10^−6^ frequency derived from *in vivo* experiments with phage M13*mp2*. In mixing experiments, DS was able to detect mutant DNA at a frequency of 1/10,000 among non-mutants. These experiments were also the source of the postulated theoretical detection limit of <10^−9^ for DS (Schmitt et al., 2012). The researchers also reported a mutant frequency of 3.5 × 10^−5^ per bp for mitochondrial DNA from human brain tissue, and this number was consistent with the results of single-molecule polymerase chain reaction and divergence rates in human pedigrees. Since then, DS has been used in diverse model systems for basic research, disease and clinical insights, and genotoxic assessments of chemicals. Table 1 and Supplementary Table S1 list the various studies and model systems that have utilized DS since its development.

**TABLE 1:** List of studies (in chronological order) where duplex sequencing (DS) was used to explore basic research questions. The organisms used and reported mutant frequencies/bp values are depicted for each study.

Number	Organism	Cell/tissue type	Mutant frequency/bp	References
1	M13 bacteriophage		2.5 × 10^−6^	Schmitt et al. (2012)
2	Human	Brain tissue (mitochondria)	3.5 × 10^−5^	Schmitt et al. (2012)
3	Human	Brain tissue (mitochondria)	Young = 3.7 × 10^−6^ Aged = 1.9 × 10^−5^	Kennedy et al. (2013)
4	Cell culture	a. Human breast epithelial stem cells b. Human breast epithelial cells (mitochondria)	1 × 10^−6^ 7.7 × 10^−7^	Ahn et al. (2015)
5	Human adenovirus 5	Entire viral genome	1.3 × 10^−7^	Risso-Ballester et al. (2016)
6	Cell culture	Human breast epithelial cells (mitochondria)	1 × 10^−5^	Ahn and Lee (2019)
7	pBR322 plasmid		No frequency	Mei et al. (2019)
8	Human	B cells isolated from blood	2.2 × 10^−4^	Shen et al. (2019)
9	*Arabidopsis thaliana*	Mitochondria and plastid	Less than 10^−7^	Wu et al. (2020)
10	Mice	a. Brain tissue b. Muscle tissue c. Oocytes (mitochondria)	4.7 × 10^−7^ 4.5 × 10^−7^ 2.4 × 10^−7^	Arbeithuber et al. (2020)
11	Endosymbiont bacteria	a. *Sulcia* b. *Nasuia*	2.04 × 10^−7^ 2.18 × 10^−5^	Waneka et al. (2021)
12	Human	Sperm (FGFR3 coding region)	6 × 10^−7^	Salazar et al. (2022)
13	*Daphnia magna*		5.6 × 10^−7^	Sobel et al. (2022)
14	*Escherichia coli*	a. Wild type (WT) b. Δ *mutL* c. Δ *mutT*	2.7 × 10^−7^ 5.3 × 10^−7^ 6.6 × 10^−7^	Bhawsinghka et al. (2023)
15	Mice	a. Brain b. Liver c. Heart (mitochondria)	2 × 10^−6^ 4 × 10^−6^ 1 × 10^−6^	Serrano et al. (2024)
16	*Arabidopsis thaliana*	Vegetative tissue a. WT b. BER^-^ c. MMR^-^	1.8 × 10^−8^ 2.6 × 10^−8^ 1.13 × 10^−6^	Waneka et al. (2024)
17	Human	a. Blood b. Spermatozoa	1.2 × 10^−7^ 2.5 × 10^−8^	Axelsson et al. (2024)
18	Human	a. Blood b. Saliva c. Oocytes (mitochondria)	1.8 × 10^−5^ 1.7 × 10^−5^ 5 × 10^−6^	Arbeithuber et al. (2025)
19	Tomato plant	Leaves	No frequency	Bonfini et al. (2025)
20	*Arabidopsis thaliana*	Mitochondria a. *radA* b. *recA3* c. *why2* Plastid a. *radA* b. *recA1* c. *osb2*	1.5 × 10^−7^ 0.8 × 10^−7^ 0.3 × 10^−7^ 0.4 × 10^−7^ 0.6 × 10^−7^ 0.1 × 10^−7^	Waneka et al. (2025)

## Basic research

The usefulness of DS in basic research has been demonstrated by several investigators, including our group. DS has been used to understand the origins and accumulation of mutations with aging and their correlations with oxidative damage (Kennedy et al., 2013; Serrano et al., 2024; Arbeithuber et al., 2020; Axelsson et al., 2024), to identify genes responsible for low mutation rates (Wu et al., 2020), and investigate the effects of transcription on mutations (Waneka et al., 2024) (Table 1). Our group showed that DS is able to detect primary DNA replication errors in a mismatch-repair deficient *E. coli mutL* strain, reproducing specific sequence contexts including leading- vs. lagging-strand error biases (Bhawsinghka et al., 2023). DNA derived from the *mutT-*deficient strain allowed detection of specific oxidative-stress-associated mutations. Table 1 lists the different systems where DS has been used for basic research along with their reported mutant frequencies.

DS is useful for identifying deleterious mutations that are unidentified by other assays, such as mutation accumulation. Mutator effects under conditions of stress or ultimate cell death, which are difficult to identify using traditional methods, can also be observed with DS by simply isolating the DNA at the desired stages. This provides information on the underlying DNA damage that caused the mutation through analysis of the SSC sequences. We note that errors made in the last round of replication can be identified in this manner before they are fixed into permanent mutations. Thus, DS enables researchers to seek basic answers to novel questions in mutagenesis, the associated mechanisms, and evolutionary consequences.

## Disease and clinical applications

Rare genetic mutations are the starting points for numerous mutation-related diseases, including cancers. Identifying mutations early on is crucial for intervention and for preventing disease progression. The mutations may also provide clinical insights for treatment and drug use, including relapses. DS has proved useful in detecting clonal and subclonal mutants in various types of cancer. Supplementary Table S1 lists the various cell and tissue types on which DS has been used to identify low-frequency mutations in diseases and cancer. For example, DS has been used to detect ovarian cancer cells in the peritoneal fluid as an indicator of malignancy (Krimmel et al., 2016) and to understand preexisting drug resistance as well as relapse in the case of chronic myeloid leukemia (Schmitt et al., 2018). The effects of chemotherapy on normal tissues can be understood, such as platinum chemotherapy in the case of pediatric cancers (Sanchez-Guixe et al., 2024). With further research on other types of cancers and biopsies, DS technology has significant potential in advancing the field of diagnostics and therapeutics. Gene-editing-based therapeutics are a kind of targeted mutagenesis that often suffer from the side effects of introducing random mutations at other sites in the genome; these can be detected by DS in addition to confirming the desired target mutations. DS is also a promising tool for identifying subclonal mutants contributing to antibiotic resistance in microorganisms and for making informed treatment choices.

## Chemical genotoxicity testing

The application of DS for genotoxicity testing was first demonstrated for aflatoxin treatment of mice (Chawanthayatham et al., 2017), which showed an order of magnitude increase in the mutation frequency in treated mice livers. Using established mutagens such as ethyl methanesulfonate, N-ethyl-N-nitrosourea, benzo[a]pyrene, and N-nitrosodimethylamine, its efficacy has been demonstrated in exposed rodents, especially over the last 2 years (Supplementary Table S1, *chemical genotoxicity*). Strong correlations were found between outcomes from DS and TGR assays, with some studies indicating higher sensitivity of DS than the TGR assay (Ashford et al., 2025; Bercu et al., 2023; LeBlanc et al., 2022; Valentine et al., 2020). However, this increased sensitivity may be attributed to the availability of larger sequence targets or favorable sequence contexts. Thus, genotoxicity assays using DS provide opportunities to reduce animal usage and assay times compared to the currently used mammalian assays. In addition, the use of diverse genomic loci may improve our understanding of the genome architecture in mutation susceptibility. A recent study by Zhang et al. (2025) showed robust reproducibility with the same DNA samples processed through library preparation and sequencing in different laboratories, further endorsing the broad applicability of DS. There are ongoing efforts to include DS in the regulatory testing of mutagens, with an expert workgroup already recommending its inclusion in future OECD guidelines (Yauk et al., 2026; Marchetti et al., 2023; Zhang et al., 2025).

## Limitations of DS

DS is theoretically capable of detecting mutation frequencies at the level of 10^−9^. However, there are some current limitations and challenges to the application of DS. At present, the practical detection limit is approximately 10^−7^ to 10^−8^; at ultralow frequencies, artifacts are generally dominant and arise due to imperfections in DNA isolation and library preparation. The damages incurred by the DNA during isolation or mechanical fragmentation serve as sites for base misincorporation. DNA end repairs after fragmentation can be erroneous and introduce mutations. Another possibility of artifact generation is amplification bias, which hinders equal amplification of both strands and results in early loss of one strand. Notably, DS is not adept at detecting larger-sized mutations, such as deletions and other complex events, for which long-read methods may be more appropriate. DS requires skilled bioinformatics analysis, which can become one of its limitations. A desired increase in detecting more mutational events along with lowering of the experimental background would require increased computational complexity. Moreover, there is a need to adopt uniform analysis parameters for comparative studies. Sequencing at great depths to obtain high-confidence DSC comes at a cost; although there is a tradeoff between increased cost and increased sensitivity, this limitation can be overcome by technological improvements.

## Perspective

Currently, several mutation detection methods are available. Of these, methods like the Ames assay were developed many years ago but continue to be used because of their simplicity, lower costs, and limited labor requirements. However, emerging technologies have enabled the development of new methods. DS is a recently developed ecNGS method with high sensitivity and capacity to detect errors as low as one among 10^9^ bases theoretically. The mutational spectra obtained from various studies indicate the high specificities of the detected mutations; such mutational footprints are valuable resources for identifying the mechanisms of mutagenesis. Deleterious mutations often escape detection but can be identified using DS. The present detection limit of DS (10^−7^ to 10^−8^) is adequate for some mutagen-induced mutations but not for measuring chromosomal mutations, which occur spontaneously at rates of 10^−9^ to 10^−11^. However, with improvements in DNA library preparation and bioinformatic approaches to filter out false positives, it should be possible to further enhance sensitivity. Thus, DS is a powerful tool whose current strengths support its important role in mutation research and potential future inclusion in regulatory testing.
