# Supplementary material for: Detection of mutations: from Ames test to duplex sequencing
Source: Front Mol Biosci. 2026 Apr 2;13:1774439. doi: 10.3389/fmolb.2026.1774439 (PMC13083020; doi:10.3389/fmolb.2026.1774439)
Supplement: Supplementary file 1 [file Table1.docx]

**Detection of mutations - from Ames test to Duplex Sequencing**

Niketa Bhawsinghka, Roel M. Schaaper

**Supplementary**

**Table S1:** Application of Duplex Sequencing technology in disease and genotoxic research (arranged chronologically by the year of publication).

|  | **Organism** | **Cell/tissue type** | **Mutant frequency/bp** | **Reference** |
| --- | --- | --- | --- | --- |
| ***Disease related*** | | | | |
| 1. | Humans | 1. Chronic Myeloid Leukemia relapse after imanitib (ABL1 gene from blood) 2. Normal prostate (pol genes) 3. Normal colon (pol genes) | 3 x 10^-7^  1.2 x 10^-7^  6.1 x 10^-8^ | (Schmitt et al., 2015) |
| 2. | Humans with ovarian cancers | Peritoneal fluid (TP53 mutations)   1. Normal 2. Cancer | 4.23 x 10^-7^  8.4 x 10^-7^ | (Krimmel et al., 2016) |
| 3. | Primary cells treated with UV | Human fibroblasts   1. Normal 2. Xeroderma pigmentosum 3. Cockayne syndrome | 8 x 10^-7^  1.7 x 10^-6^  2 x 10^-6^ | (Reid-Bayliss et al., 2016) |
| 4. | Humans with chronic myeloid leukemia | Peripheral blood  (ABL1 mutations)   1. Clinical trial of imatinib 2. Normal | 3.7 x 10^-7^  1.8 x 10^-7^ | (Schmitt et al., 2018) |
| 5. | Humans | Uterine lavage (TP53)   1. Normal 2. Ovarian cancer | 2 x 10^-6^  3 x 10^-6^ | (Salk et al., 2019) |
| 6. | Humans with Ph+ALL (Philadelphia chromosome-positive acute lymphoblastic leukemia) | Bone marrow and  Peripheral blood  (ABL1) | (2-4) x 10^-5^ | (Short et al., 2020) |
| 7. | Humans (tumors) | Plasma  Fibroblast tissue | No frequency | (Hallermayr et al., 2022) |
| 8. | Humans | Myeloid cells from sickle cell patients | 2 x 10^-4^ | (Spencer Chapman et al., 2023) |
| 9. | Humans (pediatric cancers) |  | No frequency | (Sanchez-Guixe et al., 2024) |
| 10. | Humans with Acute Myeloid Leukemia | Peripheral blood | No frequency | (Dillon et al., 2024) |
| 11. | Humans | Endometrium (TP53) | 2 x 10^-7^ | (Rios-Doria et al., 2025) |
| 12. | Humans | Buccal mucosa | No frequency | (Yokoyama et al., 2025) |
| ***Chemical genotoxicity*** | | | | |
| 13. | Mice treated with aflatoxin B1 | Liver   1. Control 2. treated | 2.7 x 10^-7^  3 x 10^-6^ | (Chawanthayatham et al., 2017) |
| 14. | Transgenic rodents   1. Big Blue Mouse 2. Tg-rasH2 | Bone marrow and liver (λ cII)   1. Treated with B[α]P 2. Treated with ENU 3. Control   Lung, spleen and blood   1. Control   b. Treated with urethane | 1.16 x 10^-6^  1.27 x 10^-6^  1.48 x 10^-7^  9.03 x 10^-8^  3 x 10^-7^ | (Valentine et al., 2020) |
| 15. | Cell culture | *In vitro* organotypic airway cultures   1. control 2. treated with EMS | 1.9 x 10^-7^  4 x 10^-7^ | (Wang et al., 2021) |
| 16. | Transgenic rodents  (MutaMouse exposed to benzo(a)pyrene ) | Bone marrow   1. control 2. treated | 1.3 x 10^-7^  1.0 x 10^-6^ | (LeBlanc et al., 2022) |
| 17. | Cell culture | Human epidermal keratinocytes (HEKa)   1. Control 2. UV radiated | 3 x 10^-6^  4 x 10^-6^ | (Zhivagui et al., 2023) |
| 18. | Mice (C57BL/6J *gpt Δ*) treated with NDMA | Lungs and liver | No frequency, only type and spectra | (Armijo et al., 2023) |
| 19. | Cell culture | Human TK6 lymphoblastoid cells a) control   1. treated with ENU | 5 x 10^-7^  1.1 x 10^-6^ | (Cho et al., 2023) |
| 20. | Transgenic rodents  (MutaMouse exposed to procarbazine hydrochloride) | Bone marrow   1. Normal 2. Treated | 1.3 x 10^-7^  3.2 x 10^-7^ | (Dodge et al., 2023) |
| 21. | Rats exposed to ENU | 1. Stomach 2. Bone marrow 3. Blood 4. Liver | 1.8 x 10^-6^  1.4 x 10^-6^  1 x 10^-6^  5 x 10^-7^ | (Smith-Roe et al., 2023) |
| 22. | Transgenic rodents  (Big Blue rats treated with NDEA) | Liver | 1.5 x 10^-6^ | (Bercu et al., 2023) |
| 23. | Fisher 344 rats | Liver   1. Untreated 2. Aristolochic acid treated 3. Methapyrilene treated | 5 x 10^-8^  2.3 x 10^-6^  5.4 x 10^-8^ | (Sahib et al., 2024) |
| 24. | Cell culture (exposed to NDMA) | 1. HepaRG 2D cells 2. HepaRG 3D spheroids | 1.87 x 10^-7^  7.2 x 10^-6^ | (Seo et al., 2024) |
| 25. | NEIL1-deficient mice treated with aflatoxin B_1_ | Liver   1. WT (unexposed) 2. WT (exposed) 3. Neil1 (unexposed) 4. Neil1 (exposed) | 6 x 10^-8^  1.3 x 10^-6^  7.4 x 10^-8^  1.5 x 10^-6^ | (Minko et al., 2024) |
| 26. | Transgenic rodents  (MutaMouse treated with BbF) | 1. Bone marrow 2. Liver | 1.5 x 10^-7^  3.6 x 10^-7^ | (Schuster et al., 2024) |
| 27. | Mice (aflatoxin exposed) | 1. WT (unexposed) 2. WT (exposed) 3. Neil1 ^-/-^ (unexposed) 4. Neil1 ^-/-^ (exposed) 5. Xpa ^-/-^ (unexposed)   Xpa ^-/-^ (exposed) | 5 x 10^-9^  2.8 x 10^-7^  4 x 10^-9^  3.5 x 10^-7^  5 x 10^-9^  3 x 10^-7^ | (Luzadder et al., 2025) |
| 28. | Transgenic rodents  (Muta Mouse treated with ENU) | Male germ cells   1. control 2. treated | 1.19 x 10^-7^  2.86 x 10^-7^ | (LeBlanc et al., 2025) |
| 29. | Cell culture (treated with formaldehyde) | *In vitro* ALI airway cultures   1. control 2. treated | 7.9 x 10^-8^  6.8 x 10^-8^ | (Le et al., 2025) |
| 30. | Transgenic rodents  (Big Blue C57BL/6 mice) | Treated with N-nitrosomorpholine   1. Liver 2. Kidney   Treated with N-nitrosoreboxetine   1. Liver | 5 x 10^-7^  2.5 x 10^-7^  8 x 10^-8^ | (Zhang et al., 2025) |
| 31. | Cell Culture (treated with 4-nitroquinoline 1-oxide) | Human TK6 lymphoblastoid cells   1. Control 2. Treated | 3.6 x 10^-7^  8.3 x 10^-7^ | (Huliganga et al., 2025) |
| 32. | Mice (treated with N-nitroso-bisoprolol) | Liver   1. untreated 2. treated   Bone marrow   1. untreated 2. treated | 9.6 x 10^-8^  2.6 x 10^-7^  6.1 x 10^-8^  1.4 x 10^-7^ | (Simon et al., 2025) |
| 33. | Transgenic rodents  (Big Blue Mouse ) | Liver   1. untreated 2. treated with NEIPA 3. treated with B[a]P | 5.3 x 10^-8^  2.5 x 10^-6^  4.6 x 10^-7^ | (Xia et al., 2025) |
| 34. | Transgenic rodents  (MutaMouse treated with BbF | Germ cells   1. untreated 2. treated | 4.4 x 10^-8^  4.7 x 10^-8^ | (Stewart et al., 2025) |
| 35. | Transgenic rodents  (Muta Mouse treated with NDMA) | Liver | 8 x 10^-7^ | (Ashford et al., 2025) |
| 36. | Cell culture (exposed to aristolochic acid) | 3D HepG2 spheroids | No frequency | (Conway et al., 2025) |

**Reference**

ARMIJO, A. L., THONGARARM, P., FEDELES, B. I., YAU, J., KAY, J. E., CORRIGAN, J. J., CHANCHAROEN, M., CHAWANTHAYATHAM, S., SAMSON, L. D., CARRASCO, S. E., ENGELWARD, B. P., FOX, J. G., CROY, R. G. & ESSIGMANN, J. M. 2023. Molecular origins of mutational spectra produced by the environmental carcinogen N-nitrosodimethylamine and S(N)1 chemotherapeutic agents. *NAR Cancer,* 5**,** zcad015.

ASHFORD, A. L., NACHMANSON, D., WILLS, J. W., HIGGINS, J. E., SMITH, T. H., VAVRA, K. C., DAHALAN, F. A., HOWE, J., ELLOWAY, J. M., SALK, J. J., DOHERTY, A. & LYNCH, A. M. 2025. Alignment between Duplex Sequencing and transgenic rodent mutation assay data in the assessment of in vivo NDMA-induced mutagenesis. *Arch Toxicol*.

BERCU, J. P., ZHANG, S., SOBOL, Z., ESCOBAR, P. A., VAN, P. & SCHULER, M. 2023. Comparison of the transgenic rodent mutation assay, error corrected next generation duplex sequencing, and the alkaline comet assay to detect dose-related mutations following exposure to N-nitrosodiethylamine. *Mutat Res Genet Toxicol Environ Mutagen,* 891**,** 503685.

CHAWANTHAYATHAM, S., VALENTINE, C. C., 3RD, FEDELES, B. I., FOX, E. J., LOEB, L. A., LEVINE, S. S., SLOCUM, S. L., WOGAN, G. N., CROY, R. G. & ESSIGMANN, J. M. 2017. Mutational spectra of aflatoxin B(1) in vivo establish biomarkers of exposure for human hepatocellular carcinoma. *Proc Natl Acad Sci U S A,* 114**,** E3101-E3109.

CHO, E., SWARTZ, C. D., WILLIAMS, A., M, V. R., RECIO, L., WITT, K. L., SCHMIDT, E. K., YAPLEE, J., SMITH, T. H., VAN, P., LO, F. Y., VALENTINE, C. C., 3RD, SALK, J. J., MARCHETTI, F., SMITH-ROE, S. L. & YAUK, C. L. 2023. Error-corrected duplex sequencing enables direct detection and quantification of mutations in human TK6 cells with strong inter-laboratory consistency. *Mutat Res Genet Toxicol Environ Mutagen,* 889**,** 503649.

CONWAY, G. E., CHAVANEL, B., VIRARD, F., SHAH, U. K., BURGUM, M. J., EVANS, S. J., KORENJAK, M., THOMAS, L. E., JENKINS, G. J., ZAVADIL, J. & DOAK, S. H. 2025. Harnessing the power of an advanced in vitro 3D liver model and error-corrected duplex sequencing for the detection of mutational signatures. *Mutagenesis*.

DILLON, L. W., HIGGINS, J., NASIF, H., OTHUS, M., BEPPU, L., SMITH, T. H., SCHMIDT, E., VALENTINE , C. I. I. I., SALK, J. J., WOOD, B. L., ERBA, H. P., RADICH, J. P. & HOURIGAN, C. S. 2024. Quantification of measurable residual disease using duplex sequencing in adults with acute myeloid leukemia. *Haematologica,* 109**,** 401-410.

DODGE, A. E., LEBLANC, D. P. M., ZHOU, G., WILLIAMS, A., MEIER, M. J., VAN, P., LO, F. Y., VALENTINE III, C. C., SALK, J. J., YAUK, C. L. & MARCHETTI, F. 2023. Duplex sequencing provides detailed characterization of mutation frequencies and spectra in the bone marrow of MutaMouse males exposed to procarbazine hydrochloride. *Arch Toxicol,* 97**,** 2245-2259.

HALLERMAYR, A., NEUHANN, T. M., STEINKE-LANGE, V., SCHARF, F., LANER, A., EWALD, R., LIESFELD, B., HOLINSKI-FEDER, E. & PICKL, J. M. A. 2022. Highly sensitive liquid biopsy Duplex sequencing complements tissue biopsy to enhance detection of clinically relevant genetic variants. *Front Oncol,* 12**,** 1014592.

HULIGANGA, E., CHO, E., SWARTZ, C. D., WILLIAMS, A., RECIO, L., SALK, J. J., MARCHETTI, F. & YAUK, C. L. 2025. Adverse Outcome Pathway-Informed Integrated Testing to Identify Chemicals Causing Genotoxicity Through Oxidative DNA Damage: Case Study on 4-Nitroquinoline 1-Oxide. *Environ Mol Mutagen,* 66**,** 185-198.

KRIMMEL, J. D., SCHMITT, M. W., HARRELL, M. I., AGNEW, K. J., KENNEDY, S. R., EMOND, M. J., LOEB, L. A., SWISHER, E. M. & RISQUES, R. A. 2016. Ultra-deep sequencing detects ovarian cancer cells in peritoneal fluid and reveals somatic TP53 mutations in noncancerous tissues. *Proc Natl Acad Sci U S A,* 113**,** 6005-10.

LE, Y., REN, B., MUSKHELISHVILI, L., DAVIS, K., WANG, Y., GWINN, W., RUA, D., HEFLICH, R. H. & CAO, X. 2025. Characterizing the Pulmonary Toxicity and Potential Mutagenicity of Formaldehyde Fumes in a Human Bronchial Epithelial Tissue Model. *Environ Mol Mutagen,* 66**,** 6-21.

LEBLANC, D. P. M., MEIER, M., LO, F. Y., SCHMIDT, E., VALENTINE, C., 3RD, WILLIAMS, A., SALK, J. J., YAUK, C. L. & MARCHETTI, F. 2022. Duplex sequencing identifies genomic features that determine susceptibility to benzo(a)pyrene-induced in vivo mutations. *BMC Genomics,* 23**,** 542.

LEBLANC, D. P. M., ZHOU, G., WILLIAMS, A., MEIER, M. J., VALENTINE, C. C., SALK, J. J., YAUK, C. L. & MARCHETTI, F. 2025. Duplex sequencing identifies unique characteristics of ENU-induced mutations in male mouse germ cellsdagger. *Biol Reprod,* 112**,** 1015-1027.

LUZADDER, M. M., MINKO, I. G., VARTANIAN, V. L., DAVENPORT, M., FEDOROV, L. M., MCCULLOUGH, A. K. & LLOYD, R. S. 2025. The Distinct Roles of NEIL1 and XPA in Limiting Aflatoxin B1-Induced Mutagenesis in Mice. *Mol Cancer Res,* 23**,** 46-58.

MINKO, I. G., LUZADDER, M. M., VARTANIAN, V. L., RICE, S. P. M., NGUYEN, M. M., SANCHEZ-CONTRERAS, M., VAN, P., KENNEDY, S. R., MCCULLOUGH, A. K. & LLOYD, R. S. 2024. Frequencies and spectra of aflatoxin B(1)-induced mutations in liver genomes of NEIL1-deficient mice as revealed by duplex sequencing. *NAR Mol Med,* 1**,** ugae006.

REID-BAYLISS, K. S., ARRON, S. T., LOEB, L. A., BEZROOKOVE, V. & CLEAVER, J. E. 2016. Why Cockayne syndrome patients do not get cancer despite their DNA repair deficiency. *Proc Natl Acad Sci U S A,* 113**,** 10151-6.

RIOS-DORIA, E., PARKER, E. U., KOHRN, B. F., PIKE, M., COOMBES, C., LATORRE-ESTEVES, E., REITER, D. J., FREDRICKSON, J., KATZ, R., SWISHER, E. M., DOLL, K. M. & RISQUES, R. A. 2025. TP53 somatic evolution in the normal endometrium of Black and White individuals. *Gynecol Oncol,* 197**,** 1-10.

SAHIB, S., YAN, J. & CHEN, T. 2024. Application of duplex sequencing to evaluate mutagenicity of aristolochic acid and methapyrilene in Fisher 344 rats. *Food Chem Toxicol,* 185**,** 114512.

SALK, J. J., LOUBET-SENEAR, K., MARITSCHNEGG, E., VALENTINE, C. C., WILLIAMS, L. N., HIGGINS, J. E., HORVAT, R., VANDERSTICHELE, A., NACHMANSON, D., BAKER, K. T., EMOND, M. J., LOTER, E., TRETIAKOVA, M., SOUSSI, T., LOEB, L. A., ZEILLINGER, R., SPEISER, P. & RISQUES, R. A. 2019. Ultra-Sensitive TP53 Sequencing for Cancer Detection Reveals Progressive Clonal Selection in Normal Tissue over a Century of Human Lifespan. *Cell Rep,* 28**,** 132-144 e3.

SANCHEZ-GUIXE, M., MUINOS, F., PINHEIRO-SANTIN, M., GONZALEZ-HUICI, V., RODRIGUEZ-HERNANDEZ, C. J., AVGUSTINOVA, A., LAVARINO, C., GONZALEZ-PEREZ, A., MORA, J. & LOPEZ-BIGAS, N. 2024. Origins of Second Malignancies in Children and Mutational Footprint of Chemotherapy in Normal Tissues. *Cancer Discov,* 14**,** 953-964.

SCHMITT, M. W., FOX, E. J., PRINDLE, M. J., REID-BAYLISS, K. S., TRUE, L. D., RADICH, J. P. & LOEB, L. A. 2015. Sequencing small genomic targets with high efficiency and extreme accuracy. *Nat Methods,* 12**,** 423-5.

SCHMITT, M. W., PRITCHARD, J. R., LEIGHOW, S. M., AMINOV, B. I., BEPPU, L., KIM, D. S., HODGSON, J. G., RIVERA, V. M., LOEB, L. A. & RADICH, J. P. 2018. Single-Molecule Sequencing Reveals Patterns of Preexisting Drug Resistance That Suggest Treatment Strategies in Philadelphia-Positive Leukemias. *Clin Cancer Res,* 24**,** 5321-5334.

SCHUSTER, D. M., LEBLANC, D. P. M., ZHOU, G., MEIER, M. J., DODGE, A. E., WHITE, P. A., LONG, A. S., WILLIAMS, A., HOBBS, C., DIESING, A., SMITH-ROE, S. L., SALK, J. J., MARCHETTI, F. & YAUK, C. L. 2024. Dose-Related Mutagenic and Clastogenic Effects of Benzo[b]fluoranthene in Mouse Somatic Tissues Detected by Duplex Sequencing and the Micronucleus Assay. *Environ Sci Technol,* 58**,** 21450-21463.

SEO, J. E., LE, Y., REVOLLO, J., MIRANDA-COLON, J., XU, H., MCKINZIE, P., MEI, N., CHEN, T., HEFLICH, R. H., ZHOU, T., ROBISON, T., BONZO, J. A. & GUO, X. 2024. Evaluating the mutagenicity of N-nitrosodimethylamine in 2D and 3D HepaRG cell cultures using error-corrected next generation sequencing. *Arch Toxicol,* 98**,** 1919-1935.

SHORT, N. J., KANTARJIAN, H., KANAGAL-SHAMANNA, R., SASAKI, K., RAVANDI, F., CORTES, J., KONOPLEVA, M., ISSA, G. C., KORNBLAU, S. M., GARCIA-MANERO, G., GARRIS, R., HIGGINS, J., PRATT, G., WILLIAMS, L. N., VALENTINE, C. C., 3RD, RIVERA, V. M., PRITCHARD, J., SALK, J. J., RADICH, J. & JABBOUR, E. 2020. Ultra-accurate Duplex Sequencing for the assessment of pretreatment ABL1 kinase domain mutations in Ph+ ALL. *Blood Cancer J,* 10**,** 61.

SIMON, S., SCHLINGEMANN, J., JOHNSON, G., BRENNEIS, C., GUESSREGEN, B., KOSTAL, J. & DIECKHOFF, J. 2025. Deriving safe limits for N-nitroso-bisoprolol by error-corrected next-generation sequencing (ecNGS) and benchmark dose (BMD) analysis, integrated with QM modeling and CYP-docking analysis. *Arch Toxicol,* 99**,** 3935-3962.

SMITH-ROE, S. L., HOBBS, C. A., HULL, V., TODD AUMAN, J., RECIO, L., STREICKER, M. A., RIVAS, M. V., PRATT, G. A., LO, F. Y., HIGGINS, J. E., SCHMIDT, E. K., WILLIAMS, L. N., NACHMANSON, D., VALENTINE III, C. C., SALK, J. J. & WITT, K. L. 2023. Adopting duplex sequencing technology for genetic toxicity testing: A proof-of-concept mutagenesis experiment with N-ethyl-N-nitrosourea (ENU)-exposed rats. *Mutat Res Genet Toxicol Environ Mutagen,* 891**,** 503669.

SPENCER CHAPMAN, M., CULL, A. H., CIUCULESCU, M. F., ESRICK, E. B., MITCHELL, E., JUNG, H., O'NEILL, L., ROBERTS, K., FABRE, M. A., WILLIAMS, N., NANGALIA, J., QUINTON, J., FOX, J. M., PELLIN, D., MAKANI, J., ARMANT, M., WILLIAMS, D. A., CAMPBELL, P. J. & KENT, D. G. 2023. Clonal selection of hematopoietic stem cells after gene therapy for sickle cell disease. *Nat Med,* 29**,** 3175-3183.

STEWART, M. T., ZHOU, G., LEBLANC, D. P. M., DODGE, A. E., MEIER, M. J., WILLIAMS, A., LONG, A. S., WHITE, P. A., YAUK, C. L. & MARCHETTI, F. 2025. CORRIGENDUM, Long Term Exposure to Benzo[b]fluoranthene Does Not Induce Mutations in MutaMouse Male Germ Cells. Reprod Toxicol, 2025, 137:108985 <http://doi.org/10.1016/j.reprotox.2025.108985>. *Reprod Toxicol,* 140**,** 109138.

VALENTINE, C. C., 3RD, YOUNG, R. R., FIELDEN, M. R., KULKARNI, R., WILLIAMS, L. N., LI, T., MINOCHERHOMJI, S. & SALK, J. J. 2020. Direct quantification of in vivo mutagenesis and carcinogenesis using duplex sequencing. *Proc Natl Acad Sci U S A,* 117**,** 33414-33425.

WANG, Y., MITTELSTAEDT, R. A., WYNNE, R., CHEN, Y., CAO, X., MUSKHELISHVILI, L., DAVIS, K., ROBISON, T. W., SUN, W., SCHMIDT, E. K., SMITH, T. H., NORGAARD, Z. K., VALENTINE, C. C., YAPLEE, J., WILLIAMS, L. N., SALK, J. J. & HEFLICH, R. H. 2021. Genetic toxicity testing using human in vitro organotypic airway cultures: Assessing DNA damage with the CometChip and mutagenesis by Duplex Sequencing. *Environ Mol Mutagen,* 62**,** 306-318.

XIA, L., YAN, J., CHEN, Y., MITCHELL, K., MCGOVERN, T. J., ATRAKCHI, A. H., HEFLICH, R. H. & CHEN, T. 2025. N-nitroso-ethylisopropylamine mutagenicity in rat liver using the cII transgenic mutation assay and duplex sequencing analysis of genomic DNA. *Chem Biol Interact,* 418**,** 111603.

YOKOYAMA, A., WATANABE, K., INOUE, Y., HIRANO, T., TAMAOKI, M., HIROHASHI, K., KAWAGUCHI, S., ISHIDA, Y., TAKEUCHI, Y., KISHIMOTO, Y., KIM, S. K., KATADA, C., NANNYA, Y., SENO, H., OGAWA, S., MUTO, M. & KAKIUCHI, N. 2025. Somatic mosaicism in the buccal mucosa reflects lifestyle and germline risk factors for esophageal squamous cell carcinoma. *Sci Transl Med,* 17**,** eadq6740.

ZHANG, S., CHEUNG, J., KOSTAL, J., VOUTCHKOVA-KOSTAL, A. & SCHULER, M. 2025. Re-Evaluating Acceptable Intake: A Comparative Study of N-Nitrosomorpholine and N-Nitroso Reboxetine Potency. *Environ Mol Mutagen,* 66**,** 80-98.

ZHIVAGUI, M., HODA, A., VALENZUELA, N., YEH, Y. Y., DAI, J., HE, Y., NANDI, S. P., OTLU, B., VAN HOUTEN, B. & ALEXANDROV, L. B. 2023. DNA damage and somatic mutations in mammalian cells after irradiation with a nail polish dryer. *Nat Commun,* 14**,** 276.
